# Microfluidics for core–shell drug carrier particles – a review

**DOI:** 10.1039/d0ra08607j

**Published:** 2020-12-23

**Authors:** Sepideh Yazdian Kashani, Amir Afzalian, Farbod Shirinichi, Mostafa Keshavarz Moraveji

**Affiliations:** Department of Chemical Engineering, Amirkabir University of Technology (Tehran Polytechnic) 1591634311 Tehran Iran moraveji@aut.ac.ir +98 21 64543182

## Abstract

Core–shell drug-carrier particles are known for their unique features. Due to the combination of superior properties not exhibited by the individual components, core–shell particles have gained a lot of interest. The structures could integrate core and shell characteristics and properties. These particles were designed for controlled drug release in the desired location. Therefore, the side effects would be minimized. So, these particles' advantages have led to the introduction of new methods and ideas for their fabrication. In the past few years, the generation of drug carrier core–shell particles in microfluidic chips has attracted much attention. This method makes it possible to produce particles at nanometer and micrometer levels of the same shape and size; it usually costs less than other methods. The other advantages of using microfluidic techniques compared to conventional bulk methods are integration capability, reproducibility, and higher efficiency. These advantages have created a positive outlook on this approach. This review gives an overview of the various fluidic concepts that are used to generate microparticles or nanoparticles. Also, an overview of traditional and more recent microfluidic devices and their design and structure for the generation of core–shell particles is given. The unique benefits of the microfluidic technique for core–shell drug carrier particle generation are demonstrated.

## Abstract 2

Microfluidics application for core–shell drug carrier particles synthesis and the advantages of using this technique compared to conventional bulk methods.

## Graphical abstract

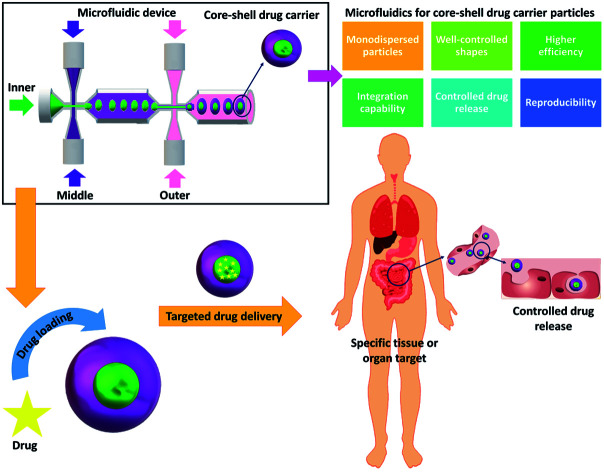

## 1. Introduction

Core–shell particles are a category of particles consisting of two or more distinct layers of material, usually a core and a shell by its name. Different or the same materials with various structures may be used for the core and the shell. This structure offers the features and properties that are not achievable by the core and shell's individual materials, providing a synergistic effect, stabilizing the active particles, and biocompatible properties.^1^

The core could be solid, liquid, or gas. The shell is typically solid, which, depending on the design requirements and the intended application, may be produced using either organic or inorganic materials.^2^ The core–shell particles can have different core shapes, shell thickness, and surface morphology.^3^

Over the past few decades, the core–shell particles have been used more frequently in drug delivery, biomedical science, tumor therapy, food and cosmetic industry, medicine, material science, and so forth according to their different properties compared to the bulk materials.^2,4,5^

Nanoparticles (NPs) synthesis is a challenging process, and due to their usage in the advanced materials, new techniques have been made for these nanoparticles' synthesis.^6^ The development of characterization techniques has led to the production of structures for these different core–shell nanocomposites.^7^

Because of these nanoparticles' different chemical and physical properties, classical physics laws would fail to explain these properties. So, some new theoretical models in nanoscales were introduced to better understand these nanomaterials. Also, the use of these nanoscales' materials leads to new advanced technologies and strategies in different fields.^8,9^

A wide variety of methods, including polymerization, spray drying, solvent evaporation, and self-assembly, have been used to prepare core–shell structures. Among these physical and chemical methods, the controllable generation of monodisperse core–shell microparticles with a narrow size distribution is of great demand. Core–shell microparticle properties such as size, morphology, and structure significantly influence their applications. It has long been a significant challenge to produce core–shell microparticles with the desired size distribution using traditional methods. These methods typically lead to high polydispersity core–shell particles. Also, they have limited control over morphology and poor reproducibility.^2^

Recently, there are many improvements in making core–shell drug carrier particles due to their specific characteristics. Microfluidics has been developed and is a promising solution for the above problems.^2,10^

## 2. Microfluidics

Microfluidics is a science of studying the fluid manipulation on micro/nanoscale, increasing the surface area-to-volume ratio.^11^ In most microfluidic devices, nanoscale fluids pass through micro-dimensional channels.^12^ In these microfluidic systems, the pressure and flow must be adjustable.^13,14^

Several kinds of materials, such as inorganic materials, metals, polymers, and plastics, are applicable for microfabrication. Microstructures and semiconductor devices have been fabricated using silicon and glass.^15–17^ Microfluidic devices are usually fabricated with polydimethylsiloxane (PDMS) polymer, glass, silicone, and polytetrafluoroethylene in the desired shape and dimensions.^18–20^

PDMS is the most common material used to fabricate microfluidic devices because of the ease of fabrication, optical transparency, gas permeability, low chemical reactivity, and inexpensiveness.^17,21,22^ It is also used in bioassays and targeted drug delivery systems (DDS) using implantable microfluidic devices.^23^ The microstructure is generally fabricated by soft lithography.^24^

Glass capillary microfluidic devices are typically made by pulling capillaries through a fine tip with an accurate size orifice, co-axially assembling them inside a wider square or circular capillary, and eventually gluing such capillaries onto a glass slide.^20^ There are a few drawbacks, considering the simplicity of glass-based microfluidics. First, the device geometry is limited, and device fabrication's technicality can be complicated, making reproducibility of devices a problem.^25^

Microfluidics is an interdisciplinary science that can be used in many scientific fields, including the pharmaceutical industry, biomedical and chemical sensors, drug delivery, cell growth, and the food industry.^26^

**Fig. 1:**
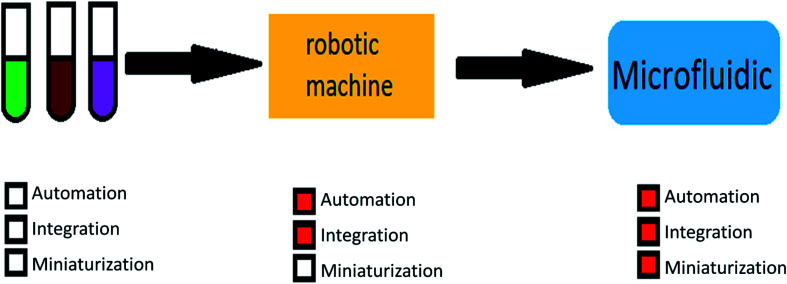
The motivation for using microfluidic devices.

The main advantages of using microfluidic systems are high efficiency, integration capability, mass production, reduced response time, and parallel operations. These mentioned properties show the importance and breadth of research in the field of microfluidic.^27–29^Fig. 1 shows the motivations for using microfluidic devices.

The presence of individual structures inside the microfluidic device, such as ducts, valves, mixers, and pumps, enables the device to allow one or more types of fluid to enter; move along the channels; if necessary, store them in a part of the chip for a while; mix them to create a specific reaction. Eventually, the original products and wastes are transferred to the outside of the device by the outputs. Also, it is worth mentioning that microfluidic systems made up of these components usually are not more than a few centimeters in size.^13,23,30,31^ All these processes can be monitored by various monitoring methods, such as optical and ultraviolet microscopes.^32,33^ In addition, the physical and chemical properties of fluids in small volumes and within capillary tubes differ from their macro-scale properties.^34,35^ In many cases, this makes it easier to work with fluids in this volume. These properties are also widely used to design chips and perform specific functions such as moving fluid inside a channel or mixing fluids.^9,36^

There is a lot of research and diagnostic tests in biology, chemistry, and medicine in which samples and soluble substances can be evaluated, so microfluidic devices have a wide range of applications in these areas. In microfluidic devices, each part of the chip can act as a part of the laboratory, and so these lab chips are called lab-on-chip.^37,38^

**Fig. 2:**
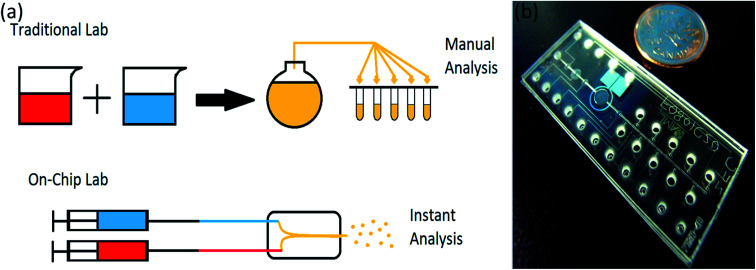
(a) Lab-on-chip in comparison with traditional lab, (b) a microfluidic chip compare to a coin, adapted from ref. 41.

A lab-on-chip is a device used instead of a laboratory process, usually on a scale of square centimeters or even millimeters in size (Fig. 2). It will do everything have to be done in a laboratory on a small device called a chip, which has become more useful in recent years.^39,40^

Another factor that has led to the use of lab-on-chips devices in recent years is the use of small amounts of fluid in these chips, which reduces costs and time of process.^42,43^ Also, the accuracy of these chips is very high. Because it is a continuous process and the operator is not involved in them, it causes less pollution to enter them and drastically reduces human errors due to high accuracy. Also, short execution time has gained a lot of attention.^10,44,45^

## 3. Drug delivery

Pharmaceutical systems use targeted transfer technology or control the release of therapeutic agents.^46^ The development of appropriate drug carriers in biomedical applications to reduce the side effects is desirable and has beneficial therapeutic effects.^47–49^ Nanoparticles are essential as drug carriers because of their ability to transport various types of drugs to different parts of the body and release them at the right time.^50,51^

Drug delivery is the mechanism or process of delivering a pharmaceutical substance that involves releasing a bioactive agent at the optimal and required rate.^51,52^

Cancer is a leading cause of death, and despite tremendous efforts to fight cancer and the existence of multiple therapy modalities, cancer treatment still is a significant problem.^53–55^ Chemotherapy is a widely used treatment for cancers, and this has led to trials for a considerable number of chemotherapeutic anticancer drugs. The biggest drawback to the clinical use of these drugs is their broad bio-distribution and short half-life. The major disadvantage of traditional drug delivery methods is their weak selectivity. Also, healthy cells are exposed to drugs' cytotoxic effects, and an inadequate portion of the applied drug arrives at the tumor position in most cases.^5,54–56^ In addition, there is a need for high drug dosage, and it is another unwanted side effect. So, new drug delivery technologies need to be promoted to solve these limitations and improve cancer therapies' potency.^5,54,57^

Recent drug delivery approaches deliver the drug to the tumor position and reduce side effects. Different types of drug delivery methods are developed for multiple healing applications. Nanoparticles are one of the most widely used carriers that, due to their ability to achieve therapeutic targets at acceptable times and doses, have a great interest in their potential in drug delivery.^5,56,58^

## 4. Core–shell particles

Core–shell particles were initially introduced to make production methods in the field of biotechnology more effective. Nowadays, with the advent of encapsulation technologies using core–shell particles, medicines can stay active for longer periods of time.^59,60^ They protect the drug against harsh conditions and provide the possibility of release under specific temperature or pH conditions; that's why these micro and nano-capsulation methods have gained more attention.^60–62^

Core-shelling can be defined as a process for trapping one substance, usually the drug, in a shell as a physical barrier to protect the core from adverse factors and conditions. The application of this method in the pharmaceutical industry has been increased recently. Also, microcapsule methods are widely used today. The application of microcapsules and nano-capsules has become very important in the pharmaceutical industry in the past few years. Therefore, a lot of research has been done in this specific field.^63,64^ The core-shelling method's properties include protection against moisture, heat, ultraviolet radiation, oxygen, and adverse conditions. Using these methods usually causes the drug to act under certain physical or chemical conditions, reducing drug usage. Also, taking these kinds of medication may reduce the drug's side effects and increase its effectiveness.^65,66^

**Fig. 3:**
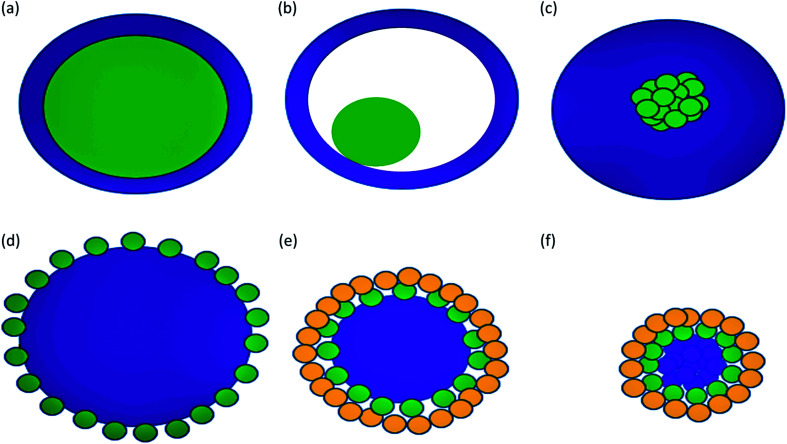
Some types of core–shell particles. The continuous shell structure (a–c): (a) single sphere core, (b) hollow shell with a small sphere inside, (c) core with the aggregation of several small spheres. (d–e) Shell with the attachment of smaller spheres onto a big core sphere. (f) Shell with the attachment of smaller spheres onto aggregated core spheres.

Some types of core–shells particles are shown in Fig. 3. Different colors are used for the core and the shell. The core may be a single sphere (Fig. 3a) or have a hollow shell with a small sphere inside, a rattle-like or yolk–shell structure (Fig. 3b). It is also possible to have an aggregation of several small spheres (Fig. 3c). The shell structure can be a continuous layer (Fig. 3a–c) or attachment of smaller spheres onto a big core sphere (Fig. 3d and e) or aggregated core spheres (Fig. 3f).^1^

### 4.1 Goals of using core–shell drug carrier particles

#### 4.1.1 Drug protection against adverse conditions

The protection of drug components can be considered as one of the primary reasons for encapsulation.^67^ The core–shell particles can maintain the active compound's stability during the synthesis, storage, and consumption stages. Shells are used as protection; core–shell drug carrier particles are produced to protect the core, most of the time the loaded drug, against destruction or releasing in undesired parts of the body.^68,69^ The drawback of interaction between substances, which may affect drugs' acceptable shelf-life, can be solved in core–shell drug carrier particles.^31,63^

#### 4.1.2 Controlled release

Core–shell particles are useful for drug delivery in the right place at the right time.^70^ This method creates an appropriate delay.^71^ There are two different types of release: delayed and stable.^56,61,65^

The first type referred to the release of active compounds with the delay in the desired location. There are many examples of delayed-release core–shell particles like encapsulated probiotics and tablets protected in the stomach against gastric acid by encapsulating them and releasing them in the small or large intestine.^72,73^ The other example of the delayed-release is using core–shell nanoparticles prepared using microfluidic chips for oral delivery of chemotherapeutics for colon cancer. They would only be released in the colon.^54^

The second type is a stable release, a mechanism designed to keep the release rate of compounds constant at the desired location. Gradually and evenly, the drug exits the shell and enters the body. The sweeteners can be encapsulated in chewing gum, which keeps the taste steady for a while.^74^

The appropriate selection of shell material for therapeutic delivery can improve controlled release, preservation, and responsiveness to stimuli.^2^

#### 4.1.3 Reduce the side effects of the drug

Most medications have several side effects.^75^ For example, chemotherapy drugs can cause hair loss, fatigue, and many other problems. When they are used in the core–shell type can significantly reduce these side effects. Because they have a delay, and the medicine would only release near the tumor, and it will not function in the whole body. The ability to release the drug only with the causative agent and only affect the causative agent and not damage other parts of the body may be considered a factor that leads scientists to work further on these core–shell drug carrier particles.^46,56,58^

### 4.2 Core–shell drug carriers release methods

#### 4.2.1 Temperature-triggered core–shell drug delivery particles

Nowadays, some polymers have specific physical properties that can be used in drug delivery. These polymers can release drugs and nanoparticles under certain conditions in the body, such as higher or lower temperatures, only in the desired areas. For example, poly-*N*-isopropyl acrylamide (PNIPAAm) polymer has unique physical properties that are used for drug delivery, including the fact that at temperatures above 32 °C, it is insoluble in water and blood. Furthermore, it is soluble in water at a temperature below 32 °C. So this polymer can be used for drug delivery to tumors near the skin surface because by cooling that area, only the drug would be released in that specific area.^61,65^ Drugs can be used as a core, and PNIPAAm can be used as a shell.

#### 4.2.2 pH-triggered core–shell particles for drug delivery

Many polymers which are used for drug delivery have specific chemical properties that make them be released only under particular pH conditions. For example, some cancer cells can be acidic or slightly basic compared to the environment and other cells, and so on. This feature can be used with a pH-sensitive shell to release the drug only where needed.^76,77^ According to previous reports, most tumors have a pH between 5.7 and 7.8, and the pH of the fluid associated with them is very rarely less than 6.5, so this feature can be used for medication. Moreover, the pH-sensitive methods have more advantages over the temperature-sensitive ones. For example, the pH-triggered core–shell particles can be useful in parts of the body that its temperature cannot be changed.^65,78^

#### 4.2.3 Sustained-release of core–shell drug carrier particles

Multiple parameters, including particle size, shell thickness, particle shape, and matrix mesh size, could control the release of drugs in microparticles by diffusion or degradation of the polymer matrix. Drugs can be progressively released from the microparticles for a specified period by tuning these parameters. The increase in particle size and shell thickness usually decreases the rate of release and prolonged duration. The encapsulation of therapeutic agents or biologically active molecules in core–shell nanostructures is a valuable technique for increasing the bioavailability of low water solubility drugs; avoiding burst releases that could cause toxicological effects, achieving sustained and extended releases; and generating temporal and spatially controlled releases.^79,80^

As mentioned in Section 4.1.2, core–shell particles could provide a controlled release of the drug. The shell may allow the core of which drug is in it, sustained-release at a steady rate, and avoid sudden release of the drug at specific parts in the human body.^2,81^

One of the most frequently employed methods to encapsulate, deliver, and release active ingredients in a sustained manner, is to trap active ingredients in polymer matrices in the shape of microspheres with a tunable degradation rate. Polylactic-*co*-glycolic acid (PLGA) is a biocompatible polymer with a degradation rate that can be tuned and is commonly used for the controlled release of drugs. However, according to the hydrophobic nature of PLGA, single PLGA microspheres are usually constrained by a low loading efficiency for hydrophilic active agents, and it remains difficult to customize their release kinetics and prevent undesired release patterns. These problems can be solved using composite microspheres with complex structures, such as core–shell composite microspheres.^82^

Deshpande *et al.*^83^ made core–shell nanogels with PNIPMAM shell and gold nanoparticles as the core for sustained and triggered release of doxorubicin (DOX).

Microparticles with a polymeric matrix with uniform size can release the encapsulated drugs more predictably. In addition, the formation of the microparticles with the porous matrix can provide more permeable sustained-release structures. In contrast, microparticle engineering with core–shell structures facilitated improved preservation. It decreased burst release, which is useful for the long-term treatment of several diseases such as asthma, angina, and psychiatric disorders.^65^

An encapsulated atorvastatin loaded porous silicon (PSi) NPs with a reactive oxygen species (ROS) was applied for diabetic wound healing.^84^ As it was mentioned, the polymeric shell formation can solve burst payload release, which is the main barrier for porous materials. The release kinetics can be easily adjusted by the shell material's choice, as the release of atorvastatin can be stimulated only by the coexistence of overproduced ROS. The release rate can be sustained for more than 24 h, making the core materials more appropriate for predicted biomedical applications.^11^

Core–shell microparticles can be obtained through evaporation of the oil from the middle layer and consolidation of the shell materials using water-in-oil-in-water (W/O/W) double emulsion with the middle oil phase containing biodegradable shell materials like PLA and PLGA as templates. For example, the PLA shell of the microparticles gradually degrades due to the hydrolysis of ester groups in the PLA chain, allowing the sustained-release of contents in the inner aqueous core.^65^

A DLPC shell and PLGA core were developed by Liu *et al.*^85^ for the sustained and controlled release of anticancer drugs with paclitaxel as a model drug. Paclitaxel's typically controlled release profile enables the use of NPs for the delivery of anticancer drugs. Sustained-release includes the ability to consistently fight cancer cells, resulting in a decline in cancer cells' viability.

Thi *et al.*^81^ showed that the sustained-release of DOX-loaded SPION@HP continuously lasted at a steady rate without an initial burst release up to 120 h, thereby retaining the therapeutic level of therapeutics for treatment over a long time.

### 4.3 Core–shell materials

The core part can be gas, liquid, or solid, and the shell is usually solid, but its nature depends on the targeted application.^86^Table 1 shows some examples of core–shell drug carrier materials, their base, the loaded drug, and their drug delivery application.

**Table 1:** Examples of core–shell drug carrier particles, their base, loaded drug, and their application^a^

Core	Shell	Base	Loaded drug	Application	Ref.
Aqueous solution	Lipid	Polymer	Doxorubicin hydrochloride and paclitaxel, anticancer drugs	Simultaneous encapsulation of synergistic actives	89
Cholesterol	Chitosan	Polymer	Paclitaxel, an anticancer drug	Encapsulation of anticancer drug for drug delivery system	112
Ferrite impregnated acrylonitrile	Acrylamide	Polymer	Naproxen, a non-steroidal anti-inflammatory drug, and trimethoprim, a bacteriostatic antibiotic	Targeted drug delivery	113
PLLA	PLGA	Polymer	Paclitaxel and suramin, anticancer drugs	Sequential and parallel drug release	114
PLGA	DLPC	Polymer	Paclitaxel, an anticancer drug	Controlled drug release	85
Oxidized sodium alginate	Chitosan	Polymer	5-Aminosalicylic acid (5-ASA), a model drug which is rapidly absorbed before entering the colon	Development of a colon-specific delivery	115
Pectin	Alginate	Polymer	Piroxicam (PRX) as a model non-steroidal anti-inflammatory drug (NSAID)	Delayed drug delivery	116
PMMA	PEI	Polymer	Ibuprofen (IB)	Intracellular drug delivery	117
PLGA	Casein	Polymer	Paclitaxel (Ptx) and epigallocatechin gallate (EGCG), anticancer drugs	Dual-drug-loaded nanomedicine	118
PLGA	Alginate	Polymer	Rifampicin	Controlled drug release	119
Fe_2_O_3_	MSN	Silica	Fluorescein sodium, an anticancer drug	Magnetically triggered multidrug release	120
AuNRs	MSN	Silica	Doxorubicin (DOX), an anticancer drug	Targeted drug delivery to cancer cells	121
PEG	MSN	Silica	GSI (γ-secretase inhibitor)	Targeted inhibition of notch signaling in breast cancer	122
Pd–Ag	MSN	Silica	Doxorubicin (DOX), an anticancer drug	Photo- and pH-triggered release of anticancer drugs	68
Au	MSN	Silica	Rhodamine B, an anticancer cargo	Controlled cargo release activated by plasmonic heating	123
UCNPs–SiO_2_	MSN	Silica	Doxorubicin (DOX), an anticancer drug	NIR-triggered anticancer drug delivery	124
Ag	Poly(*N*-isopropylacrylamide-*co*-acrylic acid)	Metal and metal oxide	Dipyridamole (DIP), an anticancer drug	pH-regulated drug delivery	125
Au	PEG	Metal and metal oxide	Temozolomide (TMZ), an anticancer drug	Optical temperature-sensing, targeted tumor cell imaging, and combined chemo-photothermal treatment	126
Ag	TiO_2_	Metal and metal oxide	Doxorubicin (DOX) and LET, anticancer drugs	Biological applications such as drug delivery	127
Fe_3_O_4_	Chitosan	Metal and metal oxide	Curcumin (Cur), an autofluorescent dye as well as an anti-tumor drug	Multimodal monitoring and drug targeting	128
Ag@SiO_2_	mTiO_2_	Metal and metal oxide	Doxorubicin (DOX), an anticancer drug	Simultaneous fluorescence-SERS bimodal imaging and drug delivery	129
Fe_3_O_4_	PMAA	Metal and metal oxide	Ceftriaxone sodium (CTX), an anti-inflammatory drug	pH-controlled drug delivery	130

a MSN = mesoporous silica-based nanoparticle.

Hollow microparticles have attracted increasing attention because of their specific properties such as higher surface area, lower density, and certain superior optical properties than bulk materials.^87^ Hollow internal microspheres are possibly used as an encapsulation vehicle to secure biologically active compounds such as proteins, enzymes, and DNA in controlled drug release.^2^

Core–shell microcapsules with an aqueous core could be applied to encapsulate and protect incompatible substances or active ingredients and drug delivery.^88^ Microparticles with an aqueous core and an oily core have appropriate space for hydrophilic and hydrophobic materials to be encapsulated in them, respectively.^65^ As can be seen in Table 1, the aqueous core can encapsulate a hydrophilic anticancer drug (doxorubicin hydrochloride). Simultaneously, the solid shell of it can encapsulate a hydrophobic anticancer drug (paclitaxel).^89^

Different materials, such as metal, metal oxides, silica, polystyrene, and polymers, could form the solid core based on their applications and production processes. One approach for producing a solid core core–shell particle is to the application of a hardcore template. Another approach is to generate core–shell particles directly by converting the emulsion droplets into solid core–shell particles, with a solid core and a solid shell. Solidification techniques include solvent evaporation, polymerization, and ionic crosslinking. A solid core structure coated with a shell layer has a great advantage for synergistic and regulated drug delivery. Although a burst release of drug is possible in a core–shell particle with a liquid core and a solid shell after the shell's breakage or degradation, the solid core structure solves this problem because it can release from the carriers only after the degradation of polymer layers.

Shell materials are commonly classified as organic and inorganic groups. There are many available materials for core and shell fabrication, and these materials specify the physical, chemical, and biological properties of them. This high versatility in the choice of core and shell materials enables core–shell microparticles with different functionalities and properties to be prepared. The shell materials can be chosen according to the application of core–shell particles. The shell also protects the core's chemically active components against corrosion, oxidative degradation, and erosion. Also, shell materials offer increased thermal stability and enhance the microparticles' electrical, optical, and magnetic properties.^2^

In the following, the most commonly used core and shell materials will be described.

#### 4.3.1 Polymer

An organic polymer or any other organic compound of high density may be used as the shell material.^2^ Polymers play one of the most prominent roles as shells in the pharmaceutical industry, whether organic polymers or natural polymers. They can have unique properties and characteristics that are very important and useful in drug delivery.^20,90^ These polymers are often used due to their unique properties, such as flexibility, optical properties, and rigidity, to target the drug and protect them against physical conditions. Examples include chitosan and poly-*N*-isopropyl acrylamide.^2,65,91,92^ Moreover, the organic shell makes it possible to obtain significant control over its cargo's permeability and biocompatibility. A metal core could be covered with an organic shell to prevent the oxidization of surface atoms into metal oxide in the presence of oxygen.^2^

There are various physically rigid polymeric materials such as polystyrene, PLGA, and isotactic polypropylene (iPP) used to produce solid core particles.^2^ As mentioned in the Section 4.2.3, PLGA has proved a useful polymer for drug delivery systems because of its high biocompatibility, biodegradability, wide range of erosion periods, and providing sustained-release of drug.^82,93^ Lukyanova *et al.*^94^ presented two microfluidic routes for producing solid-core/solid-shell particles. Poly(methyl methacrylate) (PMMA) was used as the solid core and encapsulated in the shell using a microfluidics device. Also, ethylene glycol dimethacrylate monomer was polymerized under UV light used to generate a rigid core.

#### 4.3.2 Silica

Silica is one of the materials used in the encapsulation. It can have high efficiency in drug encapsulation due to its large cavities and contact surface area, and it is very biocompatible. Also, by adding organic and inorganic materials to it, it can be given unique properties suitable for drug release.^54,56,59^ In fields such as healthcare, separation, biotechnology, and biomedical sensing, silica has a wide variety of practical applications. Chemical stability, low cost, and formability are the unique characteristics of silica that allow spherical particles from nano to micrometer size to be formed.^95^ The silica shell protects the core from coalescing and from undesirable contamination of the surrounding. Also, through a chemical reaction, silica could be modified and form a resistant and rigid shell. Silica chemical inertia may be a shielding agent which prevents the core from being degraded. Also, because silica is optically transparent, it can enhance the core's spectroscopic analysis.^2,96^

In high-performance liquid chromatography, solid silica-core/porous-shell particles could be used for the separation with a high flow rate and relatively low backpressure. The small solid core protected by the porous shell results in a larger particle and a higher surface area, resulting in lower backpressure for the separation.^1,97^

#### 4.3.3 Metals and metal oxides

Metals and their oxides are also used for encapsulation.

Compared to polymer shells, a metal shell such as zeolite, titanium, and gold acts as a more powerful barrier and prevents small molecules' undesired release into the core. Furthermore, since inorganic materials' thermal conductivity is greater than that of polymers, microparticles' thermal conductivity can be greatly enhanced by inorganic additives such as metals in the shell. There may also be other special characteristics of these materials, such as magnetic properties.^2^

Metal and metal oxides are mostly used as cores and for drug delivery. Because with the help of a magnetic field, the drug can be easily transported to the desired location. Various metals and their oxides nanomaterials are unsuitable and toxic to the human body. However, MnO, TiO_2_, and ZnO nanoparticles have been considered in drug delivery.^5,98–100^

### 4.4 Materials for improving core–shell drug carriers

#### 4.4.1 Magnetic nanomaterials

In the 1970s, Widder's theory of magnetic drug delivery was introduced.^101^ Magnetic core–shell carriers have gained a lot of interest because of their physicochemical and structural properties. Localized treatment and stability under external magnetic fields are the main aspects of magnetic nanoparticles (MNPs). In addition, to obtain a responsive property to a particular stimulus, such as pH, heat, or even enzymes, these MNPs may be coated or functionalized.^102^

The core–shell drug carrier particles produced using magnetic nanoparticles could be led to the specific neighborhoods for releasing the drug in the body using an external magnetic field. For example, after nanocarriers' injection in magnetically induced systems, an extracorporeal magnetic field is used to concentrate drug-loaded nanocarriers at tumor sites. Some suitable magnetic stimulation candidates are structured core–shell nanoparticles coated with silica, polymer, or magnetoliposome (maghemite nanocrystals encapsulated in liposomes).^103^

**Fig. 4:**
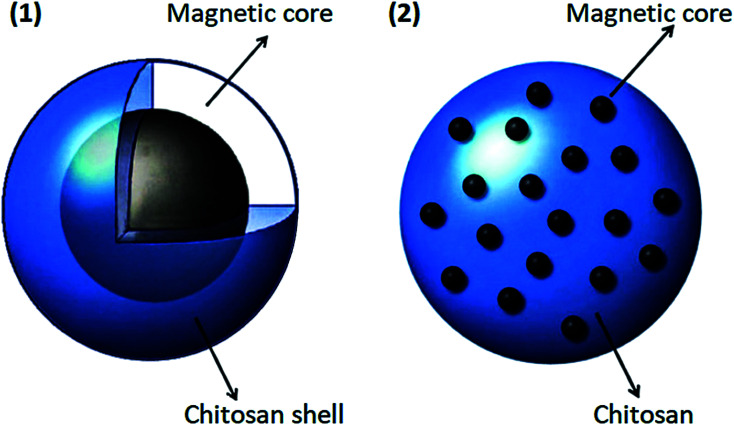
Two types of coating magnetic core in chitosan, this figure has been adapted from ref. 108 with permission from Elsevier B.V., copyright 2013.

Due to magnetic nanoparticles' instability in aqueous solutions, they cannot be used alone as drug carriers. A practical method to eliminate or minimize this problem is to use some coatings. Pharmaceutical magnetic nanoparticles with a magnetic core should be stabilized because these magnetic materials should be kept stable to use for drug delivery purposes.^104^ The magnetic core is used to release the drug to a specific location, and the polymer is used to load, transfer, and dispense the drug. In general, magnetic nanoparticles have high chemical activity and are easily oxidized in the presence of air.^104–106^ As a result, their magnetic properties will be lost. When these magnetic particles are coated with a suitable shell, they are protected from oxidation, which leads to a reduction in toxicity, and aggregation of these materials would be minimized. Also, the coating can enhance the stability of these magnetic drug carrier particles.^107^ In addition to protecting and stabilizing magnetic particles, suitable coatings can be used as a surface agent to make them more functional due to the presence of amino, hydroxyl, and carboxylate groups. One of the best coatings for this method is chitosan. Chitosan nanoparticles are one of the most promising protective coatings for magnetic nanoparticles due to their unique properties. As shown in Fig. 4, a multi-core or single-core magnetic structure can be used for chitosan nanoparticles.^108^

#### 4.4.2 Polymer–metal nanomaterials

Wang *et al.*^109^ synthesized Au-impregnated polyacrylonitrile (PAN)/polythiophene (PTH) core–shell nanofibers, which had improved semiconducting properties such as mobility. Various polymer–metal nanocomposites are made of silver, gold, platinum, palladium, *etc.*^3^ The nanocomposite preparation is achieved by adding a metal precursor to the polymer solution and reducing the metal material's precipitation.^5,110^

Silver nanoparticles have antibacterial properties, and this feature is the basis of their application.^110^ Mixing silver nanoparticles with polymer and making nanocomposites is one of their most-used application method.^5,111^

Silver nanoparticles with anti-fungal and anti-inflammatory effects are environmentally friendly, non-thermal, heat-resistant, and highly corrosion-resistant. As an example, in 2015, hydrogel granules of chitosan/silver nanocomposite were produced in the presence of NaBH_4_ as a reducing agent.^5,111^

Gold nanomaterials have attracted a great deal of attention in biomedical applications due to their high biocompatibility, low toxicity, and relatively low reactivity. They tend to accumulate in various forms of rods and prisms during synthesis. They have significant applications as sensors, solar cells, and in the field of pharmaceutical and tissue engineering. Gold nanoparticles and chitosan bound to glycolic acid were reported to be synthesized for pharmaceutical and engineering applications.^131,132^

While copper and its compounds have antibacterial properties already known in ancient times, they currently receive renewed attention due to copper's possible use in healthcare situations as an antibacterial material.^133^

Compared to other metals, copper is less toxic to bio cells and has a higher potency than micronutrients. Although copper nanoparticles are known for their unique characteristics, there is not much research on these nanoparticles. Anticancer activity of various metals such as Cu, Si, Se, Zn, Ag has been reported. In the meantime, copper has become more widespread due to its unique and excellent electronic configuration. Participation as a significant factor in the oxidation–reduction cycle of enzymes is one of copper's interesting properties. The anticancer role of copper compounds such as CuO, CuS has been reported in some research.^5,131,132^

The antibacterial effect of novel core–shell nanostructures based on copper and silver metals against *Escherichia coli* (*E. coli*) has been investigated. These nanostructures were prepared separately using the non-toxic, biodegradable, and biocompatible biopolymers chitosan and guar gum–polyvinyl alcohol (GG–PVA). A well-diffusion approach against *E. coli* analyzed the antibacterial property of the core–shell nanostructures. Due to the high ratio of NZVCu in the nanostructure, Cu/CuO@SiO_2_ nanostructures are very effective against *E. coli*.^134^

Also, recently, Fe_3_O_4_@copper(ii) metal–organic framework Cu_3_(BTC)_2_ (Cu-BTC) as core–shell structured magnetic microspheres were investigated. The slowly released copper ions and improved production of reactive oxygen species (ROS) played a role in Fe_3_O_4_@Cu-BTC antibacterial activity by promoting the successful isolation and transfer of photoexcited electron–hole pairs.^135^

#### 4.4.3 Carbon nanomaterials

Research on carbon nanomaterials containing carbon nanotubes, graphite, graphene, *etc.*, is considered because of their unique mechanical properties. Carbon-based core–shell (CBCS) materials can provide rapid interfacial transport at various porous length scales due to their porosity mimicking natural systems, the high surface area for reactions, and strong dispersion of active sites, and decrease the diffusion effect or shorten the diffusion pathways that can be effectively utilized in energy storage.^136^ So, the advantages of both materials can be taken by producing composite materials from carbon and natural polymer. For example, graphene oxide is a two-dimensional nanomaterial made from natural graphite.^137–139^ This material has a low-density capability, a very high electrical conductivity, and excellent strength. Recently, the application of graphene oxide in biomedical fields, especially pharmaceuticals, has become widespread due to attractive properties such as large surface area and excellent stability in water. Changes in the level of graphene oxide are significant for effective release to achieve appropriate drug loading and biocompatibility.^138,140,141^

Meanwhile, chitosan polymer is a naturally occurring cationic polycarbonate with better biocompatibility and degradability properties than other cationic polymers. Compared to graphene oxide (GO), graphene oxide–chitosan (GO–CS) nanocomposites are smaller in size, positively charged, and less toxic. As an example, it was reported that the CpG oligodeoxynucleotides (ODNs) delivery system based on GO–CS nanocomposites significantly improved the loading capacity and cellular uptake. Therefore to increase the delivery efficiency of CpG ODNs, GO–CS nanocomposites will serve as efficient nanocarriers.^140,141^

#### 4.4.4 Amines

Some researchers have suggested that adding some particular amines to the surface of the core–shell nanoparticles can cause the shell to be more inclined to that specific cell's surface than other cells. Alternatively, it may go away, and this can lead to a more controlled drug release. This property allows amines to be used to improve drug release and drug delivery performance.^65,142,143^

In another research, the use of amine-containing core–shell nanoparticles has been studied as possible drug carriers for intracellular delivery. Thick poly(ethyleneimine) PEI shells (approximately 30 nm) greatly improved the drug loading potential of the complexed nanoparticle up to 23% (w/w).^117^

Also, silanes are available by different amine groups and can improve the functionality of the magnetite nanoparticle surface for protein conjugation. Therefore, silane-coated MNPs result in high-quality materials for magnetic drug delivery systems.^5^

## 5. Microfluidics devices in drug delivery systems

Microparticles with controllable structures are needed to be capable of quantitative encapsulation of drugs and regulated release of them to the desired location to ensure reduced side effects and optimized therapeutic efficacy. The traditional delivery methods, including oral, intravenous, sublingual, and intramuscular drug deliveries, have drawbacks, such as interactions with foods, low solubility and permeability, and irregular absorption, making steady-state dosing difficult to achieve in patients. Many drugs are also toxic to normal tissues or toxic when overdosed, but they are useless when underdosed. Consequently, control of the amount of encapsulation and drug release rate is needed. The fabrication of uniform microparticles with controllable and flexible sizes, compositions, and internal structures is needed to fulfill these demands.^65,144^ Most traditional bulk drug synthesis methods suffer from many drawbacks, such as the need to use a high quantity of valuable drugs or chemicals, the generation of polydisperse particles that influence the release profile, the limitation of the generation of multiple therapeutic agent-loaded carriers, and the difficulty associated with locating the delivery of drugs and *in vivo* investigation of the therapeutic or toxic effects that needs many animals.^50^

New technologies, like microfluidics, can solve these problems. For the production of effective drug carrier particles, microfluidic devices offer specific advantages. Compared to bulk methods, microfluidic technology allows the production of highly stable, uniform, monodispersed particles with higher encapsulation efficiency by effectively regulating the fabricated chip's geometries and the flow rates of multiphase fluids.^50,144^

In traditional bulk synthesis methods, both the inertial and viscous effects govern mass transport in fluids, associated with nonlinearities that give rise to numerous instabilities, such as turbulence. In contrast, the inertial effect becomes negligible in microfluidics. This feature allows microfluidics to synthesize nanoparticles in a highly regulated and reproducible manner that has been difficult to achieve in traditional macroscale methods.^68,123,145^ Microfluidics-templated emulsions facilitate the controlled drug release by producing highly uniform microparticles with well-controlled sizes, shapes, and compositions.^65^

Microfluidics can be used for polymer synthesis with precise forms or chemicals for drug delivery application. For example, a technique developed by Nie *et al.*^146^ to use the capillary instability-driven break-up of a liquid jet made up of two immiscible fluids for producing polymer particles with various shapes and morphologies. The stated strategy allows the emulsification process to be precisely controlled, leading to monodispersed droplets with controlled morphologies ranging from 20 to 200 μm in size.^144^

In addition, some conventional delivery methods, including painful and harmful injections, can benefit from microscale technologies by fabrication of microneedles or needle-free injection devices. In order to improve the comfort and quality of life of patients, microfluidic systems have recently been developed for transdermal administration of drugs.^51^

Also, microfluidics' drug delivery systems have improved drug encapsulation efficiency, allowing the use of two or more drug molecules in the same carrier for combination therapy or dual function.^12^

Microfluidic systems can be used for the direct delivery of active molecules, in addition to the potential of producing complex drug carriers.^147^ In order to maximize the local availability of the drug and reduce the side effects induced by the drug's interaction with other organs and tissues, such systems are capable of efficiently transporting drugs to a targeted location. Furthermore, for so-called transdermal delivery, which is direct drug delivery through the skin, microfluidic systems have been successfully used. These systems, which utilize a needle or an array of microneedles, transfer the drug across the skin (epidermis) barrier.^51^

Recent advances in developing and utilizing such platforms for drug delivery systems have been discussed elsewhere^12,20,49–51,65,144,145^ are not reviewed here. In the following section, we will focus on core–shell drug carrier particles production in microfluidic devices.

In addition to basic solid microparticles, microfluidic devices offer a flexible approach to producing more complex functional microparticles with core–shell particles with multicompartmental structures for co-encapsulation and synergistic release of specific drugs. Also, they provide highly efficient encapsulation and regulated release of hydrophilic or hydrophobic drugs and. For biomedical and pharmaceutical applications, diverse controlled release types, such as sustained-release, triggered release, and combined release of both release styles can be accomplished based on their advanced shell functions and internal structures.^65^

By their name, core–shell particles are a category of particles that contain a core and a shell. Different materials or the same materials with different structures can be used as the core and the shell. Due to the combination of superior properties not exhibited by the individual components, core–shell particles have gained a lot of interest. The structures could integrate core and shell characteristics and properties, where the surface properties of the shell are passed to the core, bringing new features to the core–shell particles.^86^

Core–shell particles are typically synthesized by a two-step or multi-step process. First, the core particles are synthesized, and, based on the type of core and shell materials and their morphologies, the shell is then formed on the core particle through various methods.^1^ In the following section, we will describe microfluidic devices' application for core–shell drug carrier particle production and discuss various microfluidic systems that have been utilized for core–shell drug carrier particle preparation, characterization, and application.

## 6. Microfluidic devices for core–shell drug carrier particles preparation, characterization, and application

Microfluidics' adaptation to the development of micro-sized structures has become more important since the first theoretical work was carried out more than 10 years ago. The primary reasons for this technology's adoption originate from the emulsion homogeneity and the high degree of control over the operation. Because of the fluids' properties in the microfluidic channels, control over the entire production chain is possible. Therefore, these systems might be used for the production of single or double emulsions.^11,12,20,148^

In recent years, a lot of research has been done about the application of microfluidic devices for making core–shell particles used in drug delivery. It is possible to use only a small proportion of samples in these microstructures, and controlled composition is one of the other significant advantages of this method. In microfluidic devices, drug-loaded particles' volume and permeability can be controlled more straightforwardly than other methods, making microfluidics more suitable for the fabrication of pharmaceutical drug carrier particles. So far, many microfluidic chips have been designed for the formation of core–shell nanoparticles drug carriers.^24,32,63,142^

The microfluidic method significantly reduces material costs and improves drug encapsulation efficiency. The most important advantage of using microfluidic devices is uniform core–shell droplets size. The polydispersity index (PDI) of these particles is usually less than 1%, which can be adjusted by the fluids' physical properties.^20^ Core–shell particles for drug delivery application are mainly fabricated by droplet microfluidics.^12,20,49–51,65,144,145^

### 6.1 Droplet microfluidics for core–shell drug carrier particles preparation

Droplet microfluidics has been increasingly developed in recent years. It allows precise control of droplet creation, encapsulations, and release kinetics using nonlinear channel geometries, such as T-junctions, co-flowing, and flow-focusing constrictions.^149^ A single emulsion is a droplet of one liquid dispersed in an immiscible fluid that forms the continuous phase.^25^ The common single emulsion includes oil-in-water (O/W) and water-in-oil (W/O) emulsions.^20^

It is complicated to make core–shell droplets made of more than one immiscible liquid by other possible methods. However, it is possible to make these multi emulsion core–shell droplets more easily in the droplet-based microfluidic devices.^5,150,151^ The droplets can be generated from a single emulsion of two partially miscible or immiscible fluids or multiple emulsions (mostly the double emulsion) of three or more immiscible fluids.^11,12,149^ The single emulsion does not guarantee that multiple therapeutics are loaded simultaneously, and it is more complicated where the payloads have different solubility. Thus in drug delivery systems, double emulsions are also commonly used.^20^

Usually, if multi emulsion core–shell droplets are needed, a sequential process of producing a single emulsion should be continued to place the primary emulsified droplet in the secondary droplet.^49,152^ Double and multiple emulsions are frequently produced by a combination of two or more flow geometries.^5^ The most common geometries in 2D are T- and Y-junctions, while for the 3D design, flow-focusing, co-flow, and various combinations of the previous designs used for the production of double emulsions.^11^

The vital point in droplet-based microfluidics is that the two liquids must either be partially miscible or immiscible. If they are miscible in each other, the core–shell droplets that will carry the drug cannot be produced.

**Fig. 5:**
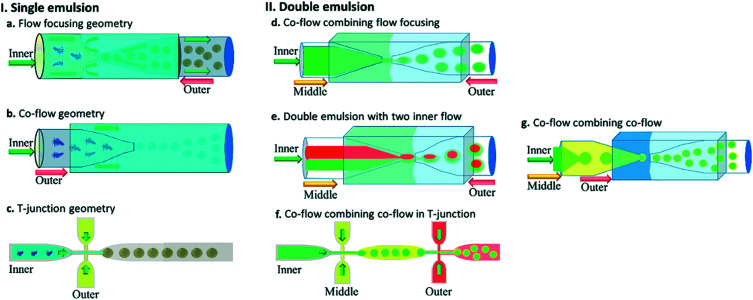
Different geometries of droplet-based microfluidic devices, this figure has been reproduced from ref. 20 with permission from Royal Society of Chemistry, copyright 2017. (a–c) The geometries for the preparation of single emulsions, (a) flow-focusing, (b) co-flow, and (c) T-junction (d–g) complex geometries to fabricate double emulsions, (d) co-flow combined with flow-focusing with one inner fluid, (e) co-flow combined with flow-focusing with two inner fluid (f) sequential T-junction, and (g) sequential co-flows for thin shell capsule production.

Fig. 5 shows different common geometries of droplet-based microfluidic devices for producing single and double emulsion.

We can classify microfluidic methods into single-step and sequential methods for double or multiple emulsion particle fabrications despite the different geometries of droplet microfluidic devices. First, we will introduce important dimensionless numbers associated with droplet-microfluidics. Then we will describe different geometries of droplet-based microfluidics, and then we will discuss single-step and sequential methods for core–shell particle preparation.

#### 6.1.1 Dimensionless numbers

The most important dimensionless numbers in droplet microfluidics are Reynolds number (Re), Péclet number (Pe), Weber number (We), and capillary number (Ca) (eqn (1)–(4) respectively).1
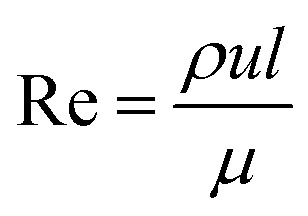
2
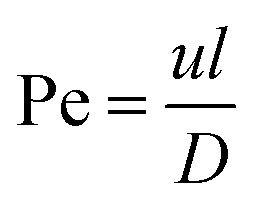
3
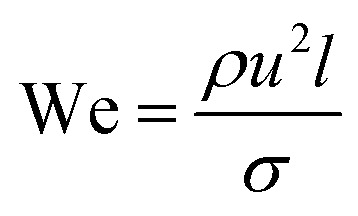
4
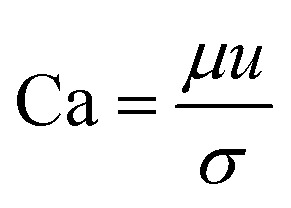
In these equations, *u* is the fluid's velocity, *l* is the characteristic length, *ρ* is the fluid's density, *μ* is the fluid's viscosity, *σ* is the surface tension, and *D* is the mass diffusion coefficient.

**Fig. 6:**
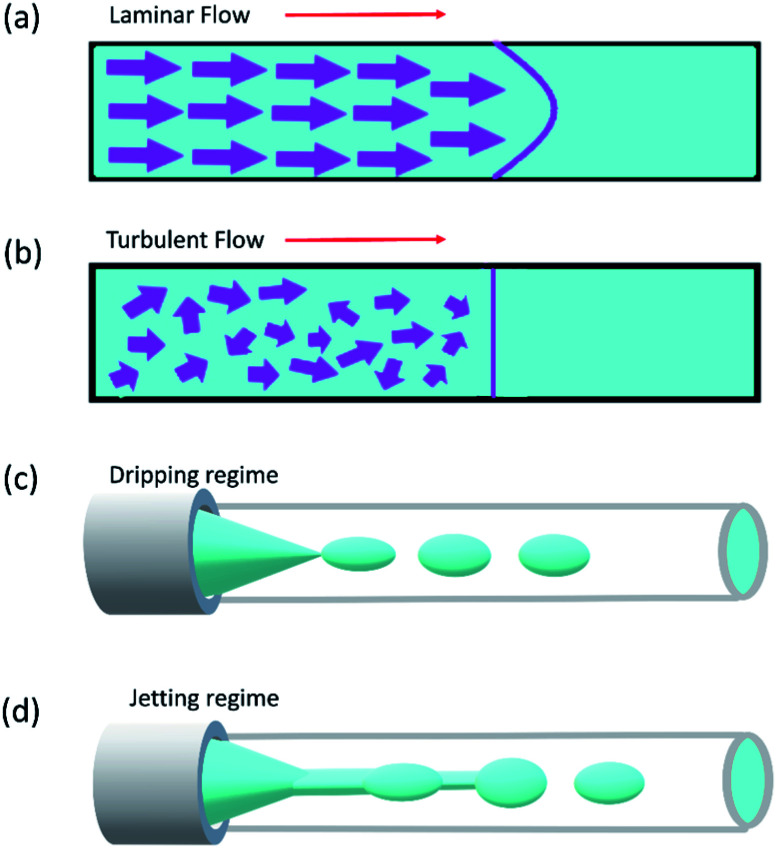
(a) Laminar flow, (b) turbulent flow, (c) dripping regime, and (d) Jetting regime.

Reynolds number and Pe reflect the fluid flow characteristics and molecules' action within the fluid.^20^ The Reynolds number describes the relationship between inertial forces and viscous forces. A flow pattern of different streamlines that are parallel to the fluid direction (Fig. 6a) is reflected in the laminar flow (usually Re number < 1800). On the other hand, the fluid flow with a high Reynolds number (usually >2300) is categorized as turbulent flow, providing a chaotic pattern without distinct streamlines (Fig. 6b). Steady streamlines characterize the laminar flow, so the fluids and molecules within the fluids can be accurately manipulated to produce controllable and monodisperse droplets, reflecting the desirable characteristics of drug encapsulation in droplet microfluidics.^11,20^

The Péclet number is a commonly applied dimensionless number for mass transfer processes, reflects the diffusion or convection of molecules in the fluids.^11,20^ Because of the small volumes and the laminar flow pattern, the transfer of molecules is slow in droplet microfluidics, primarily by diffusion instead of convection. Thus droplet microfluidics minimizes the transition of molecules from the dispersed phase to the continuous phase, allowing the high efficiency of drug encapsulation into the droplets.^20^

Two forms of instability in the fluid's behavior in a droplet-based microfluidic device are described: dripping and jetting regimen (Fig. 6c and d). The transition from one regimen to the other happens by increasing the flow rate and dimensionless parameters (*e.g.*, capillary and Weber numbers).^12^ The Weber number, defined by eqn (3), is an essential descriptor of deformation in the droplets. The Weber number determines the relationship between surface tension and inertial forces. With the increase in deformation, this number increases; higher energy is therefore meant to generate smaller emulsions.^11,12^

The capillary number described by eqn (4) expresses the ratio between the viscous forces over the fluid's surface tension. In droplet-based microfluidics, the capillary number is of particular importance since it enables the investigation of various break-up patterns identified by different capillary number ranges. In the case of low values of the capillary number (range <10^−2^), the droplet formation is not influenced by the shear stress. In order to form a thread and finally squeeze out the droplet, it is only dependent on the accumulated pressure in the inner channel. According to Rayleigh–Plateau instability, droplet formation will occur when the droplet's maximum extension is greater than 1.^11^

Dripping and jetting regimen happens when the inner fluid is pumped into the secondary immiscible fluid, like in the formation of single emulsion droplets. Parameters related to fluids such as their viscosities, surface tensions, densities, flow rate, and the ratio between flow rates and the device characteristics such as geometry and surface chemistry control the formation of the droplet.^153^ The two-fluid system's behavior can be expressed according to the outer fluid's capillary number and the inner one's Weber number, *i.e.*, the outer fluid's viscous forces and the inner one's inertial forces. Monodispersed drops result from dripping instability since the device disruptions that lead to drop formation are insensitive to any external intervention, whereas the jetting regimen creates polydispersed drops. The processes leading to the formation of the drops are identical in the case of double emulsions. The fluid stream breaks simultaneously when the inner and middle fluids' rates are equal, leading to the creation of a double emulsion presenting one internal drop. The outer fluid flow rate governs the transitions in the double or multiple emulsion devices between the two regimes; dripping happens while the outer fluid is slower, and *vice versa* for jetting.^12^

As mentioned, co-flow, flow-focusing, and T-junction, under a dripping or jetting regimen, are the most common geometries in droplet microfluidics. The junction shape defines the interface between the two immiscible flows. The droplet will be generated when the drag force is higher than the viscose force.^20^ We can control the droplet formation by understanding these theories, and droplet sizes can be perfectly controlled with passive methods by tuning the microfluidic device geometry, interfacial tension and viscosity of liquid phases, flow rates, and pressure, or with active methods by electrical forces, magnetic force, temperature, and acoustic force.^5,12,143,154,155^

#### 6.1.2 Flow-focusing geometry

The dispersed phase and continuous phase flow through two sides of the channel in flow-focusing droplet microfluidic devices and meet before the inner capillary orifice. The droplets are formed at the orifice (Fig. 5a). The physical mechanisms in the flow-focusing geometry are complicated, and in a device presenting such geometry, both dripping and jetting regimes can be created.^20,156^ When fluids with different velocities are introduced side by side, hydrodynamic focusing evolves. Hydrodynamic flow-focusing systems are generally easy to manufacture and operate and capable of generating particles with a uniform size distribution. Flow-focusing particles are usually <1 μm, which is too small for long-term payload release applications.^25,145^

#### 6.1.3 Co-flowing geometry

Two co-axially aligned fluids move in the same direction in a co-flow system. So, two capillaries have to be aligned co-axially.^25^ The co-flow geometry describes a structure in which the dispersed phase in the inner capillary flows in the same direction through an orifice of specified dimension into a continuous flow from the outer capillary (Fig. 5b). The droplets are mainly formed due to Rayleigh–Plateau instability in this configuration; thus, the jetting regime is often involved.^20,156^ In the industry, some fittings allow you to do this, but they are expensive. Using a cylindrical capillary for the inner liquid and a capillary with a square cross-section for the outer fluid is a cheaper way. Naturally, alignment is accomplished by matching the outer diameter of the cylindrical capillary's circular cross-section and the inner side of the outer one's square cross-section.^25^

#### 6.1.4 T-junction geometry

The simplest and most used microfluidic geometry is T-junction (Fig. 5c). The orthogonal channel in standard geometry includes the dispersed phase that intersects the main channel, which is filled with the continuous phase and droplets synthesized by cross-flow at the channel intersection.^20,30,49,152,157^ Oil-in-water (O/W) emulsions or water-in-oil-in-water (W/O/W) double emulsion can be generated in hydrophilic channels. In contrast, water-in-oil (W/O) or oil-in-water-in-oil (O/W/O) emulsions can be generated in hydrophobic channels.^158,159^ In this geometry, the droplet size can be regulated either actively or passively. Active control can be modulated using external actuation such as magnetically or pneumatically actuated micro-valves and integrated micro-heaters. In contrast, passive control can be regulated by flow rate controlling.^50,160^ The advantage of T-junction is to produce better monodisperse droplets.^20^

A Y-junction has the same process, but only the branches' angle makes it different from a T-junction. Also, by adding more branches to these specific designs, they can produce multi emulsion core–shell particles.^161–163^

#### 6.1.5 Single-step method

As it was mentioned in the development of microparticles for drug delivery, all three described geometries and a combination of them have been used to generate double and multiple emulsions. Double emulsions are typically generated using a combination of two geometries (Fig. 5d–g).^20^

By its name single-step droplet formation method has only one step.^164^ A conventional double emulsion microfluidic device in a single-step method is made of two round capillaries with the orifices facing each other where the round capillaries are inserted into a square capillary (Fig. 5d). The inner phase goes through the inner capillary. In contrast, the middle and outer phases in the same and opposite direction of the inner flow go through the outer square capillary. This can be regarded as a co-flow that incorporates flow-focusing geometry. Once the three fluids enter the inner capillary facing the other inner inlet capillary, which operates as a collection tube, the double emulsion is formed. Water-in-oil-in-water (W/O/W) and oil-in-water-in-oil (O/W/O) are included in the double emulsions. In the production of drug delivery microcapsules with a hollow core and a polymer shell, the W/O/W emulsion has been commonly used. The inner and outer droplets' size can be controlled by adjusting the flow rates and ratios of the inner, middle, and continuous phases, thus controlling the size and shell thickness of the generated microcapsules. The double emulsion drops' size is mainly controlled by the continuous phase flow rate in the dripping mode, while the middle layer determines the shell thickness. Thus the greater the flow ratio between the inner and middle phases, the thicker the capsule layer, for a set flow rate of the continuous phase.^165^ The multiple-component emulsions can be produced by incorporating two inner fluids simultaneously (Fig. 5e).^166^

**Fig. 7:**
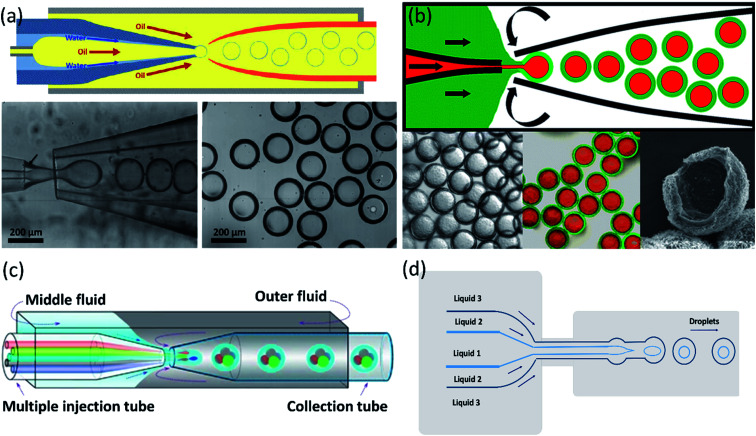
Single-step methods for a double-emulsion generation. (a) Schematic of the microfluidic device for generation of O/W/O double-emulsion droplet with an ultra-thin shell. The bottom images are optical microscope images showing the droplets in the dripping regime and the generated monodisperse double-emulsion droplets, this figure has been adapted from ref. 165 with permission from Royal Society of Chemistry, copyright 2011. (b) A microcapillary device used for the generation of double emulsion droplets for making core–shell particles. The bottom images are microscopy images of core–shell particles, fluorescence detection of the encapsulated drugs: paclitaxel (green) encapsulated within the lipid shell, and doxorubicin (red) in the liquid core, and electron microscopy image of a cracked lipid shell, respectively from left to right, this figure has been adapted from ref. 89 with permission from American Chemical Society, copyright 2013. (c) A schematic of the capillary microfluidic device used for the generation of the multiple core double emulsions, this figure has been adapted from ref. 167 with permission from Springer Nature Limited, copyright 2012, licensed under the Creative Commons Attribution-NonCommercial-No Derivative Works 3.0 Unported License. (d) Schematic of 2D flow-focusing microfluidic device used to generate double emulsion droplets in a single-step method, this figure has been reproduced from ref. 146 with permission from American Chemical Society, copyright 2005.

Kim *et al.*^165^ introduced a single-step emulsification method that creates monodisperse double-emulsion drops in a core–shell geometry with an ultra-thin wall as a middle layer using a co-axial capillary microfluidic device (Fig. 7a). A circular capillary with the tapered tip was inserted co-axially into a larger capillary and fixed into another square tube. A rounded capillary was located on the other side of the square capillary to limit the injection tip's exit flow. The aqueous fluid and oil, respectively, flow along the capillary wall and from the central capillary. The core–shell structured emulsion with a very thin shell with a thickness of 100 nanometers or even less was generated at the outlet of the injection tube after solidification. Despite the small shell thickness, these particles are very stable and have great encapsulation ability. It was demonstrated by developing biodegradable poly(lactic acid) microcapsules with a shell thickness of about 10 nanometers, potentially useful for drug delivery.

For synergistic combinations of drugs in therapeutic applications, simultaneous encapsulation of multiple active substances in a single carrier is important. However, conventional carrier systems frequently lack efficient encapsulation and release of integrated substances, mainly when drug combinations must be released at concentrations of a specified ratio. Windbergs *et al.*^89^ introduced a novel biodegradable core–shell carrier system produced in a solvent-free single-step microfluidic device. The aqueous core is encapsulated with a hydrophilic drug (doxorubicin hydrochloride), while the solid shell is encapsulated with a hydrophobic drug (paclitaxel). In this process, it is possible to control the particle size and composition precisely. Also, core–shell drug-carrier particles can be dried and stored as a powder. Two tapered cylindrical capillary tubes nested inside a square capillary whose inner dimensions correspond to the cylindrical capillaries' outer diameters were used in the microfluidic device (Fig. 7b).

A multiple-core double emulsion composed of multiple oil cores was developed by Zhao *et al.*^167^ using a glass capillary microfluidic system with multiple injection tubes (Fig. 7c). There are five separate internal channels in the injection tube, allowing four different oil phase fluids (indicated in red, green, blue, and gray colors) and one aqueous phase fluid (indicated in the center of the four oil fluids as cyan color) to enter the devices separately. The multiple core double emulsion was used as a template to create the photonic crystal barcodes. Barcodes are made of polyethylene glycol (PEG) hydrogel shells and multiple photonic crystals or magnetic-tagged ethoxylated trimethylolpropane triacrylate (ETPTA) cores. Under magnetic fields, the presence of magnetism in the barcodes provides their controllable motion. So they have a unique characteristic that makes them a perfect choice as encoded microcarriers for biomedical applications.

In addition to 3D and capillary microfluidic devices used in the single-step method for producing core–shell particles, 2D devices can also be used. Nie *et al.*^146^ presented a 2D flow-focusing microfluidic device to produce core–shell droplets in a single-step method. A double flow-focusing unit in this system forces three immiscible fluids into an orifice and then forms droplets in the downstream chamber (Fig. 7d).

#### 6.1.6 Sequential method

The general procedure for preparing double emulsions is the sequential process. Two consecutive steps in sequential methods generate emulsions of oil-in-water-in-oil (O/W/O) and water-in-oil-in-water (W/O/W). First, the inner droplets form, and then the shells' outer layers are set around the core. Then, using methods such as solvent evaporation,^168^ photo or thermally induced free-radical polymerization,^169^ ionic crosslinking,^170^ and freezing,^89^ solidification of the core–shell droplets can result in core–shell microcapsules. The sequential method usually combines two geometry. The core droplets are generated in the first geometry and the outer droplet, which encapsulates the core generated by the second geometry.^20^ Two flow-focusing, co-flowing, two T-junction, and two cross-flowing, and so on can be used in sequential method.^171,172^ The production frequency of the droplets, the size of the core, the thickness of the shell, and even the number of cores encapsulated in the shell can be precisely modulated by changing the flow rates of the fluids and the geometry of the microfluidic device.^171,173^

Through a sequential process in two steps (Fig. 5f), a double emulsion may be formed by T-junction geometry. The inner fluid is encapsulated in the first step by the middle fluid, creating a single emulsion. The droplets then flow into the second drop maker and are encapsulated, and the double emulsions are produced by the outer fluid.^20^

The basis for the generation of core–shell droplets is the same in both 2D and 3D devices,^171^ except that 3D devices remove the wettability constraints imposed by 2D devices. The droplets in 3D microfluidic systems have minimum interaction with the channel wall in comparison to 2D devices. This ability will prevent the fragile shell from rupturing and the channels from wetting during early interfacial polymerization.^2,164^

A microfluidic double emulsion geometry (Fig. 5g) has been developed to obtain very thin shells: the inner capillary is placed into a middle capillary. The middle capillary is then placed into the square outer capillary facing the collection capillary orifice within the outer capillary. In this case, the inner, middle, and outer phases flow through multiple capillaries but in the same direction, combining co-flow and co-flow geometry.^165^

**Fig. 8:**
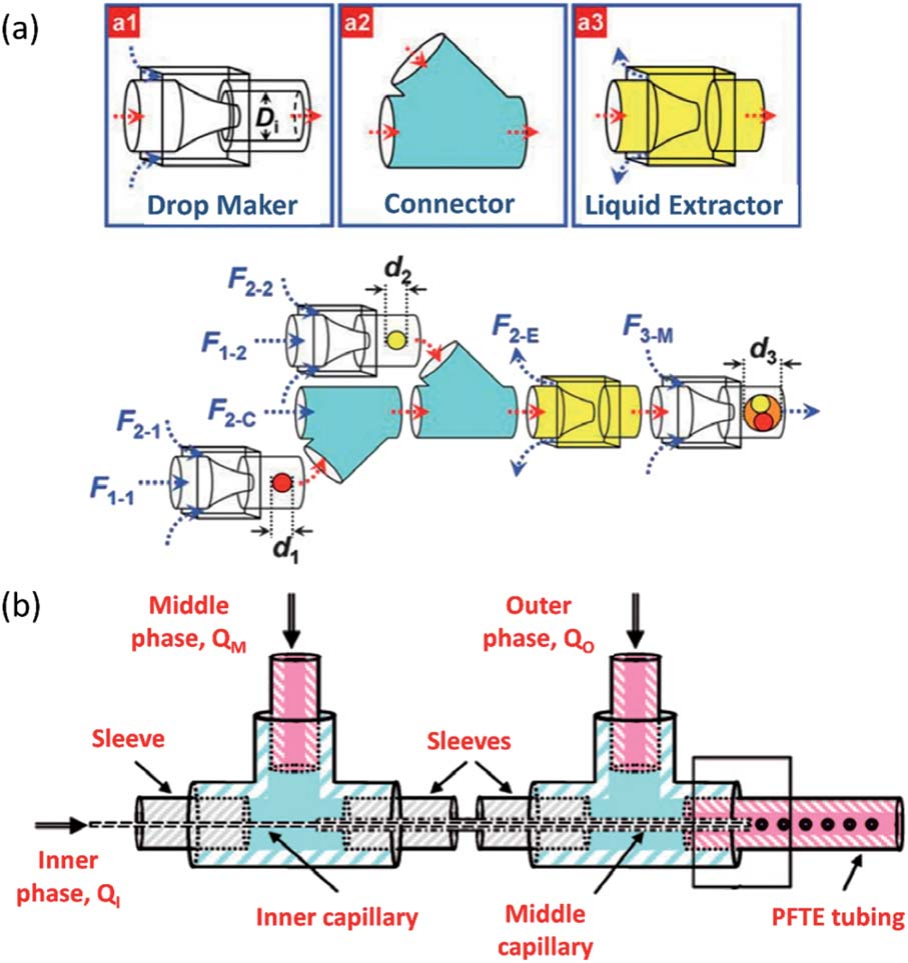
Sequential methods for a double-emulsion generation (a) microfluidic device for the controlled generation of quadruple-component double emulsions and its functional building blocks, this figure has been adapted from ref. 174 with permission from Royal Society of Chemistry, copyright 2011. (b) Schematic of the various co-axial capillaries microfluidic devices for the polymer core–polymer shell particles production, this figure has been adapted from ref. 175 with permission from Royal Society of Chemistry, copyright 2009.

Wang *et al.*^174^ developed a hierarchical and flexible microfluidic device fabricated from a combination of three building blocks, including a drop maker, a connector, and a liquid extractor that allows multiple emulsions to be strongly controlled by multicomponent generation (Fig. 8a). Droplets are made in the drop maker and then merged using the connectors. The liquid extractor removes excess continuous phase. The size, number, and ratio of the co-encapsulated droplets could be precisely tuned. This combination also enables the scale-up of the device to produce higher-order multicomponent multiple emulsions with extremely different structures. These multicomponent multiple emulsions offer a flexible and promising system for synergistic delivery of precisely encapsulated incompatible actives or chemicals.

Chang *et al.*^175^ presented a 3D microfluidic device with two co-axial capillaries for the polymer core–polymer shell particles' tuneable generation in two sequential steps (Fig. 8b). As can be seen, the device consists of two capillaries with hydrophilic or hydrophobic inner walls co-axially placed inside a T-junction along its main axis. The core and shell droplets were generated in the inner and middle capillaries, respectively, and core–shell droplets then were dispersed in an outer continuous aqueous phase.

Development in the microfluidic field is not limited to the creation of liquid-in-liquid-in-liquid (L/L/L) microemulsion; researchers have demonstrated the generation of emulsions of gas-in-water-in-oil (G/W/O) and gas-in-oil-in-water (G/O/W). In preparing G/W/O and G/O/W emulsions that could be used as a template for producing hollow microparticles, this technology has shown great advantages. A few academic researchers have attempted to use various devices such as flow-focusing, double flow-focusing, co-flowing, T-junction, and dual-coaxial geometry to prepare G/L/L emulsion.^2^

### 6.2 Recent advances of microfluidic devices for core–shell particles preparation

**Fig. 9:**
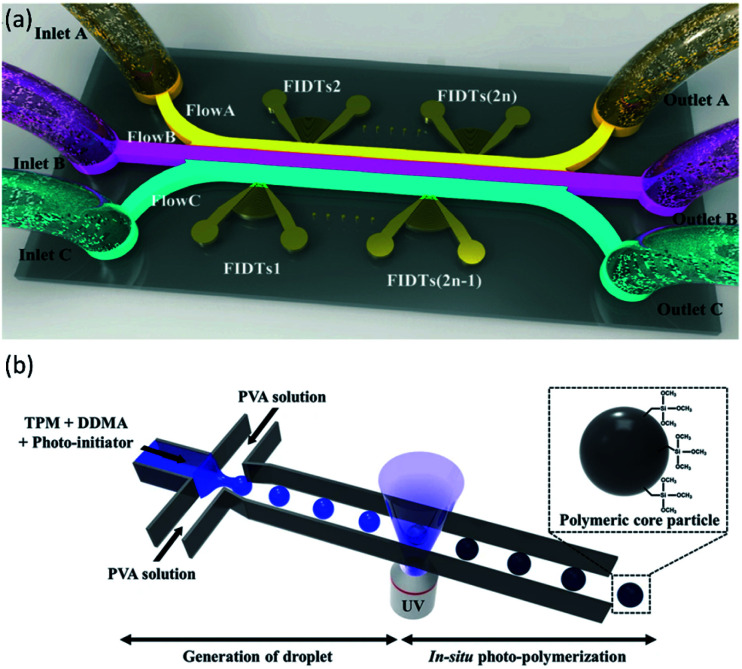
(a) Schematic of FSAW-based microfluidic device to generate core–shell microcapsules, this figure has been adapted from ref. 176 with permission from Royal Society of Chemistry, copyright 2020. (b) Schematic of monodisperse hybrid particle production, a shear force guided pinch-off mechanism shapes droplets containing the monomers (TPM and DDMA) and the photoinitiator in the continuous process (PVA solution). Photopolymerization is initiated by UV-irradiation, this figure has been reproduced from ref. 177 with permission from Springer Nature Limited, copyright 2018, licensed under a Creative Commons Attribution 4.0 International License.

In addition to common microfluidic devices for the fabrication of core–shell particles, some complicated microstructures have been introduced recently. In order to produce microcapsules with a core–shell structure, Jin *et al.*^176^ showed the application of focused surface acoustic wave (FSAW) microfluidics with one or two focused interdigital transducers (FIDTs) and a bonded polydimethylsiloxane (PDMS) microfluidic channel on a lithium niobate (LiNbO_3_) substrate (Fig. 9a). The FIDTs are placed on both sides of the flow channel to generate opposing FSAWs. It drives the particles back and forth across the oil/water interface, ideal for generating solid core–shell microcapsules and coating an aqueous microdroplet core with the oil shells. In comparison with previous methods, including T-junction, flow-focusing, co-flowing microfluidic devices, and pillar-based microfluidic devices, solid particles or liquid microdroplets in multiphase laminar flow are generated by the acoustic radiation force resulting from the FSAW without any special modification using one or two FIDTs on a microfluidic device with a simple configuration. This work provides a new active technique for producing a structure of the core–shell on the solid particles. More FIDTs can be added to the device to create more microcapsule layers if necessary. Thus it is possible to synthesize single-layer, two-layer, or even multi-layer microcapsules as desired.^176^

Kim *et al.*^177^ proposed a microfluidic method for the fabrication of organic–inorganic hybrid core–shell microparticles in which the core is from poly(1,10-decanediol dimethacrylate-*co*-trimethoxysilyl propyl methacrylate) (P(DDMA-*co*-TPM)) shell is from silica nanoparticles. In this method, in combination with *in situ* photopolymerization, the droplet-based microfluidic method generates highly monodisperse organic microparticles from P(DDMA-*co*-TPM) in a simple way (Fig. 9b). The silica nanoparticles gradually develop on the surface of the microparticles prepared by hydrolysis and tetraethoxysilane (TEOS) condensation in a simple ammonium hydroxide medium without excessive surface treatment. This approach leads to a decrease in the number of processes and, compared to traditional approaches, facilitates significantly improved size uniformity.^177^

Ahrberg *et al.*^178^ demonstrated an automated microfluidic capillary droplet reactor for the multi-step iron oxide/gold core–shell nanoparticles synthesis. Synthesis outcomes can be monitored in real-time by incorporating a transmission measurement at the outlet of the reactor.

Recently a novel fabrication technique for core–shell structure nanoparticles was created by combining microfluidic chip and electrohydrodynamic atomization to resolve the drawbacks of drug-loaded nanoparticles, such as high initial burst release and wide size distribution. The mixture solution of the surfactant (1,2-dipalmitoyl-*sn-glycero*-3-phosphoglycerol) and the polymeric coating material (polylactic-glycolic-acid) was injected into the microfluidic chip's outer microchannel as the shell of the particle in this experiment. The encapsulated drug (paclitaxel) was injected into the inner microchannel as the core. Then by applying an electric field on the laminar flow that was developed in the microfluidic chip, the particles with a nanoscale-sized core–shell structure were created. The drug release of these nanoparticles may be extended for more than ten days over a considerable period of time. It can be anticipated that this innovative technology will provide a useful platform for the production of drug-loaded core–shell nanoparticles.^179^

### 6.3 Microfluidic devices for core–shell drug carrier particles application in drug delivery

Core–shell drug-carrier particles are known for their unique features. Due to the combination of superior properties not exhibited by the individual components, core–shell particles have gained a lot of interest. As drug delivery carriers, core–shell particles have many benefits, including the reduction in initial burst release, sustained and controlled drug release rate, and the ability to carry a wide variety of biomolecules.^2^ Core–shell particles could be used in cancer treatment because they could encapsulate multiple ingredients and release them during a multistage process. In contrast, core–shell particles with a broad size distribution produced by conventional techniques are not appropriate for drug delivery.^180^ The core–shell droplets generated using the microfluidics technique allow for high encapsulation and loading efficiency. The microparticles have a narrow size distribution profile, uniform morphology, and composition resulting in a steady and controlled drug release. The microparticle size is an essential factor in selecting a suitable drug delivery method.^80^ For example, microparticles with a size distribution from a few to hundreds of microns are more appropriate for oral drug delivery.^181^

Li *et al.*^10^ produced a new form of core–shell particles for synergistic and sustained drug delivery. They were made from gelatin methacrylate (GelMa) aqueous solution as core and PLGA oil solution as shell in which different hydrophilic and hydrophobic drugs, such as doxorubicin hydrochloride (DOX) and camptothecin (CPT) could be loaded, respectively. Since the inner cores were polymerized in the microfluidics when the double emulsions were generated, the hydrophilic actives could be trapped with high efficiency in the cores. During the solidification of the microparticle shells with other actives, the cores' rupture or fusion could be avoided. During microfluidic emulsification, the microparticles' size and components can be easily and precisely controlled by adjusting the flow solutions. The encapsulated actives were only released from the delivery systems with the degradation of the biopolymer layers due to the solid nature of the resulting microparticles. Thus the burst release of the actives was prevented. These characteristics of the microparticles make them suitable for drug delivery applications.^2^

Xu *et al.*^182^ developed a doxorubicin-loaded core–shell structured microsphere through a coaxial electro-hydrodynamic atomization process. A PLGA core and a poly(d,l-lactic acid) (PDLLA) shell were included in the microspheres. As a model drug, doxorubicin, a hydrophilic chemotherapy drug, was used and encapsulated within the core. Doxorubicin was effectively encapsulated and lead to an approximately drug-free shell. Doxorubicin release was a two-stage operation, with a steady rate of release.

Nie *et al.*^180^ also used the same approach for the development of a distinct core–shell structure of microparticles. In a single step, two different hydrophilic drugs were encapsulated in microparticles with enhanced encapsulation efficiency. They showed that different drug release profiles were affected by varying the outer and inner flow ratio. In addition, the performance of different microspheres in cytotoxicity, cellular apoptosis were analyzed *in vitro*. Also, tumor inhibition against subcutaneous U87 glioma xenograft was performed *in vivo*. The benefits and potential applications of this kind of multi-drug release system in the treatment of brain tumors were demonstrated.

Ho *et al.*^183^ defined a flexible technique for developing monodisperse polymersomes with biocompatible and biodegradable diblock copolymers for the efficient encapsulation of active substances, which is a good demonstration of core–shell droplets where a dewetting transition occurred. Due to osmotic shock, the release was triggered. The double emulsion droplets were used as a template to generate PEG–PLA polymersomes by using amphiphilic diblock copolymers. The polymersomes were used for the encapsulation of a hydrophilic fluorescent solution. An osmotic pressure difference caused the polymersomes' breakage by adding salt, and the solutes were released. This easy and efficient release mechanism could be used to design encapsulation and controlled release in various biomedical applications.

### 6.4 Microfluidic devices for core–shell drug carrier particles characterization

Size, shape, drug loading, and stability are the most important properties of nanoparticles to be characterized before probing their interaction with biological systems. Also, the development of novel particle characterization tools for drug delivery greatly affects the probability of an effective therapeutic translation. An inability to verify the drug delivery system's safety *in vivo* is one real obstacle to the clinical-scale application of nanoparticles.^145^ This section presents microfluidic techniques capable of characterizing drug delivery nanoparticles.

#### 6.4.1 Size and morphology characterization

The most fundamental feature of drug delivery nanoparticles is particle size, a major determinant of bio-distribution and retention in target tissues.^184^ For particle size determination, dynamic light scattering (DLS) measurements are widely used. DLS can calculate the size of particles in suspension, according to the Stokes–Einstein equation.^145^ Microfluidic devices can create a platform for real-time *in situ* monitoring of nanoparticle formation, allowing the fundamental reaction processes of nanoparticle synthesis to be investigated. To optimize nanoscale particle production, the investigation of mechanisms behind nucleation and growth is important. The synthesis of cysteine-capped quantum dot nanocrystals between two interdiffusing reagent streams was investigated in a continuous flow microfluidic device in one platform using spatially resolved photoluminescence spectroscopy and imaging.^185^ In addition, to demonstrate the kinetics and mechanisms of nanoparticle nucleation and growth during synthesis in a microfluidic channel, small angle X-ray scattering (SAXS) was used. Although recent developments in label-free particle characterization, particle detection remains a challenge for integrating high-throughput microfluidic technologies. So size and morphology characterization of particles can be assessed using advanced microscopic techniques such as atomic force microscopy (AFM), scanning electron microscopy (SEM), transmission electron microscopy (TEM), and DLS.^145^

#### 6.4.2 Drug loading and release characterization

The behavior of drug release is a key parameter for the application of nanoparticles and is directly related to drug stability and therapeutic effects. The rate of drug release usually depends on (1) the solubility of the drug, (2) the desorption of the surface-bound or adsorbed drug, (3) the diffusion of the drug out of the nanomaterial matrix, (4) the erosion or degradation of the nanomaterial matrix, and (5) the combination of the mechanism of erosion and diffusion. Therefore, it is important to evaluate the degree of the release of the drug and obtain such knowledge that most release methods involve the separation of the drug and its delivery vehicle. The particles' drug loading ability is generally defined as the amount of drug bound per carrier mass. This parameter is calculated by various traditional techniques, such as UV spectroscopy or high-performance liquid chromatography (HPLC) and gel filtration.^145^

Microfluidic-based liquid chromatography (LC) has attracted a lot of attention because of its improved sensitivity, reduced sample utilization, and ability to multiplex measurements. Gao *et al.*^186^ developed an integrated microfluidic device with mass spectrometry (MS) detection for high-throughput drug screening. A concentration gradient generator, cell culture chamber, and solid-phase extraction columns were incorporated into this microfluidic device into a PDMS chip. The method of drug absorption and cytotoxicity assessment may be achieved simultaneously with the use of combination systems.

With a low amount of reagent usage, integrated devices may provide a means of high-throughput drug analysis. In addition, many microfluidic-based nanospray emitters^187,188^ and microfluidic-based LC-MS analysis^189,190^ have been introduced.

## 7. Conclusions

In this review, various kinds of core–shell microparticles are first discussed based on the core and shell structures' materials. The motivation of using core–shell particles is to combine the desired properties of different materials and structures to offer a synergistic effect, stabilize the active particles, or provide biocompatible properties. In addition, using core–shell drug carrier particles aims to reduce the drug's side effects with protection against environmental conditions, deliver it to the desired location, reduce its production cost, and increase its efficacy and controlled release. The choice of shell material and sometimes core for pharmaceutical systems is very complicated and should be chosen depending on the conditions. For example, suppose the desired area for releasing the drug is close to the skin's surface. In that case, it is possible to use a shell destroyed by the temperature. It is possible to direct it to the target position using a magnetic field if metal or metal oxide is used.

This review also provides an overview of microfluidic techniques for the generation of core–shell drug carrier particles. Conventional methods have some drawbacks which have considerably limited their applications. The main disadvantages of these methods are low monodispersity and high material usage. Microfluidic devices have been developed to generate core–shell particles with controlled features and providing many advantages such as cheapness, uniformity of particle size and shape, and simplicity of application compared to the other alternatives. Different microfluidic chip designs can be used based on the desired type of core–shell drug carrier particle, so there are more alternatives than the other available methods. Microfluidic devices are classified based on their geometry, and they could be designed to generate core–shell particles using single-step or sequential emulsification methods.

On the other hand, nanoparticles can be easily made in nanoscale by applying microfluidic devices, which is very difficult or impossible to achieve in the other methods. In some cases, particles made with microfluidic devices reach a few hundred nanometers in diameter, making this method popular. The microfluidic method has some more advantages, such as automation, integration, miniaturization, and the possibility of reducing human error. Their coherence makes these chips the best choice for the pharmaceutical industry. All of the above is evidence that microfluidic chips are the perfect choice for making core–shell drug carriers particle.

## Conflicts of interest

There are no conflicts to declare.

## Supplementary Material
